# Good eutrophication status is a challenging goal for coastal waters

**DOI:** 10.1007/s13280-023-01965-7

**Published:** 2023-12-23

**Authors:** Kari Hyytiäinen, Inese Huttunen, Niina Kotamäki, Harri Kuosa, Janne Ropponen

**Affiliations:** 1 Department of Economics and Management, University of Helsinki, P.O. Box 27, 00014 Helsinki, Finland; 2 Marine and Freshwater Solutions, Finnish Environment Institute, Latokartanonkaari 11, 00790 Helsinki, Finland; 3 Marine and Freshwater Solutions, Finnish Environment Institute, Survontie 9 A, 40500 Jyväskylä, Finland

**Keywords:** Eutrophication, Explorative scenarios, Integrated modeling, Simulation, Target-seeking scenarios

## Abstract

Our objective is to understand the effectiveness of local and international nutrient pollution mitigation efforts when targeting better water quality in the region’s coastal waters. To this end, we developed an integrated modeling framework for the Archipelago Sea located in the Baltic Sea in Northern Europe, conducted what-if analyses for various ambition levels of nutrient abatement, and studied the long-term consequences at the sea basin scale. We demonstrate that in outer parts of the Archipelago Sea, a good eutrophication status can be achieved if the current internationally agreed policy goals for nutrient abatement are successfully met. In inner coastal areas, current goals for phytoplankton biomass could be reached only through extreme mitigation efforts in all polluting sectors and large-scale application of yet poorly tested ecological engineering methods. This result calls for carefully considering the relevance of current threshold values for phytoplankton and its role as a dominant indicator of good ecological status.

**Supplementary Information:**

The online version contains supplementary material available at 10.1007/s13280-023-01965-7.

## Introduction

Coastal ecosystems are hotspots for contrasting ambitions: demand for amenity services, food supply, and marine protection, and the necessity of using coastal waters as vectors and storage for marine debris, nutrients, and other pollutants. These unique and heterogenous ecosystems are threatened by multiple human pressures that jeopardize the resilience of plant and animal communities accustomed to conditions at the border between sea and land (Lotze et al. 2006; He and Silliman 2019). Coastal pollution diminishes opportunities for water and beach recreation, results in economic losses for businesses in the blue economy, and poses health risks to humans, domestic animals, and coastal flora and fauna. Coastal waters, classified as territorial waters, fall under the jurisdiction of the coastal state. Consequently, each sovereign nation bears the responsibility of developing environmental legislation, policies, and institutions necessary for safeguarding its coastal waters. However, many pollutants, including chemicals, microplastics, and nutrients, readily dissolve in water, disperse, and become diluted across expansive bodies of water. They can also be transported over long distances by marine currents. Consequently, pollution originating in other countries and regions further impacts the condition of coastal waters, compounding the pollution stemming from local sources such as cities, industries, and agricultural land located on or near the coast or within the watershed.

To what extent can local communities and regions manage the eutrophication status of their adjacent coastal ecosystems? How much do coastal water conditions depend on pollution levels and mitigation efforts happening in other areas? How do local water conservation practices, along with actions taken in other locations, impact the timing and geographical spread of pollution-induced damages? These are questions addressed in this paper. As an example of coastal pollution, we study the possibilities of combating eutrophication, which is a pervasive problem in the Baltic Sea (Andersen et al. 2017), Chesapeake Bay (Kemp et al. 2005), Gulf of Mexico (Rabalais et al. 2007), Black Sea (Kideys 2002), Adriatic Sea (Justić 1987), and many other coastal regions globally. Eutrophication results from the excessive influx of nutrients (nitrogen and phosphorus) into aquatic environments from various sources, including catchment areas, industries, and households (Anderson et al. 2002). Effective measures to combat eutrophication encompass the enhancement of sewage treatment and other point-source technologies to reduce nutrient discharge, as well as the implementation of management practices aimed at curtailing nutrient runoff from agricultural and forested areas.

**Fig. 1:**
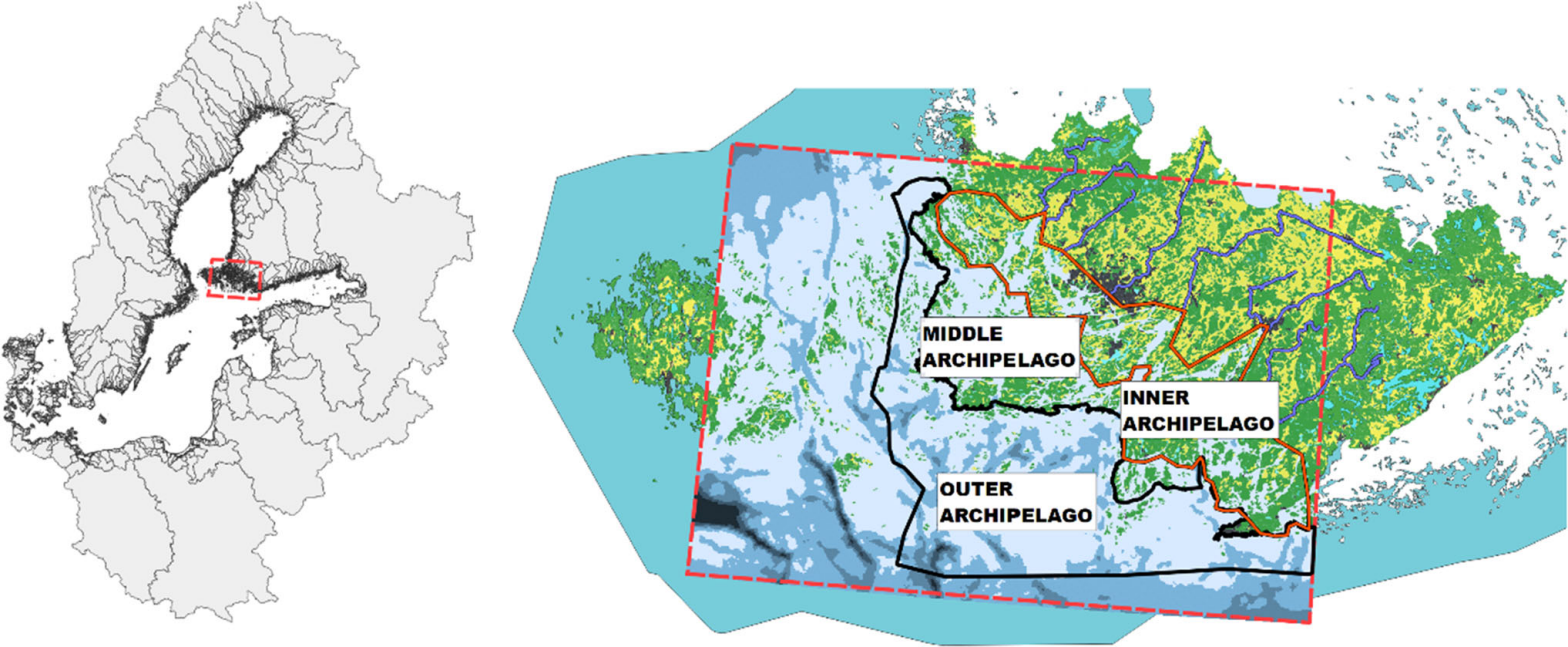
A map of the case study area. Left: the Archipelago Sea (dashed rectangle) is part of the Baltic Sea—a marginal sea of the Atlantic located in Northern Europe. The Archipelago Sea is located between the Gulf of Bothnia, the Gulf of Finland, and the Sea of Åland, within Finnish territorial waters. Right: The Archipelago Sea including the modeled marine area (dashed rectangle with a depth map) and its catchment area. Land use in the catchment: green denotes forests, yellow denotes agricultural land, and black denotes built land. The largest rivers are included as dark blue lines

Our case study area is the Archipelago Sea, which is a globally unique formation of numerous tightly clustered small islands located in the northern part of Europe, belonging to the nation of Finland, and making part of the Baltic Sea (Fig. 1). The health of coastal ecosystems in the area is driven both by nutrients transported from other parts of the Baltic Sea and by nutrients draining to the Archipelago Sea from its own catchment area in southwestern Finland. The catchment area of the Archipelago Sea is dominated by intensive agriculture and animal husbandry, and only a few lakes slow down the nutrient leakage from highly erodible soils. The Archipelago Sea is highly demanded and appreciated as a location for residence (year-round homes and summer cottages), recreation, and boating, and it also has high potential for blue nature-based tourism (Bonsdorff et al. 1997). Although nutrient inputs into the Baltic Sea have substantially reduced over the past three decades compared to the peak levels seen in the 1980s (Gustafsson et al. 2012), further reductions of phosphorus and nitrogen would be needed to meet the targets of internationally agreed water protection goal: the Baltic Sea Action Plan (HELCOM 2021). During the period between 2010 and 2019, the average levels of phosphorus and nitrogen entering the Archipelago Sea from its own catchment area exceed the national target by 7% and 4%, respectively. However, to offset the impacts of changing climate on nutrient loading, it has been estimated that a 25% reduction in phosphorus load and a 10% reduction in nitrogen load is needed for the rivers draining into the Archipelago Sea (Laamanen et al. 2021).

The policy context is the national implementation of the EU’s Water Framework Directive (WFD) (EU 2000) which requires controlling anthropogenic pressures to a degree that allows for reaching good ecological (and chemical) status (GES) in coastal waters, consisting of inner, middle, and outer archipelago (Fig. 1). The Archipelago Sea also includes western Åland Archipelago that is also under the WFD, but it is left out from this assessment. In addition, an ambitious program by the Finnish Government aims to reduce the diffuse load in the Archipelago Sea catchment area to a level that makes it possible to reach a good healthy state in Archipelago Sea and to remove the catchment area by 2027 from the HELCOM hotspot list of the worst polluters in the region (Laurila et al. 2021). It is justified to ask whether this goal is reachable and realistic by unilateral efforts conducted in the catchment area only and what level of progress should happen in neighboring areas.

The Baltic Sea is a semi-enclosed and young sea. Its geography was formed by the last glacier period, and the sea area is still under change due to a strong uplift of land (Eronen et al. 2001). The ecosystem consists of a unique combination of plant and animal species adapted to northern conditions and brackish water of varying salt concentrations (Reusch et al. 2018). The drivers and processes causing eutrophication are relatively well understood (Gustafsson et al. 2012; Andersen et al. 2017; Snoeijs-Leijonmalm et al. 2017). Future loads are conditional to several societal drivers (e.g., population growth and changes in consumer values), technological developments in polluting sectors, and changing climate (Pihlainen et al. 2020). Researchers have examined the consequences of climate scenarios, as demonstrated, e.g., by Meier et al. (2021) and have integrated them with socioeconomic scenarios in studies by Bartosova et al. (2019) and Pihlainen et al. (2020) to gain insights into the future conditions of the Baltic Sea over the long term. These studies show the consequences of alternative global scenarios on water quality parameters at high spatial resolution, but they do not address varying nutrient mitigation efforts across regions sharing the coastline and the catchment area of the sea. Some economic studies investigating the stability of international environmental agreements (Markowska and Żylicz 1999; Ahlvik and Pavlova 2013) and the cost-effective combination of measures (Ahlvik et al. 2014; Czajkowski et al. 2021) allow flexible and varying nutrient abatement efforts across regions and countries, but they do not detail and elaborate the consequences in terms of the spatial distribution of improvements or decreases in water quality or the provision of aquatic ecosystem services.

The objective of our study is to understand the impacts and effectiveness of unilateral local vs. multilateral international pollution mitigation efforts when targeting better water quality in the region’s own nearby coastal waters. For this end, we develop a modeling framework to describe human–nature interactions in the Archipelago Sea and conduct what-if analyses for future societal development and policy effort in nutrient abatement. The framework extends upon the methodology employed in previous research conducted by, e.g., Booth et al. (2016), Olesen et al. (2019) and Huttunen et al. (2021). We extend the simulations for nine scenarios of nutrient loading including varying levels of water protection efforts at the catchment areas draining to the Archipelago Sea, and other catchment areas draining to the Baltic Sea. Our broader aim is to develop and demonstrate a spatially and temporally explicit modeling framework to study the adequacy of planned water protection efforts and relevance of the existing indicators of ecological status.

## Materials and methods

### Scenarios

**Fig. 2:**
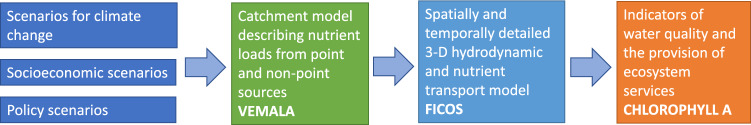
Modeling framework

To explore the plausible future ecological qualities of the Archipelago Sea consists of four steps (Fig. 2). At first, a set of exploratory and target-seeking scenarios addressing the research question and reflecting alternative future conditions in the society, climate, and policy environment are chosen. We extend the computations to nine scenarios (Table 1) including different levels of mitigation effort, technological development, and societal development, both in the catchment area of the Archipelago Sea and in other regions sharing the Baltic Sea catchment. Scenario A is a business-as-usual (BAU) scenario assuming current nutrient loading will remain in future. Scenarios B–D are target-seeking scenarios that show the consequences of reaching current internationally agreed water protection goal: the Baltic Sea Action Plan (BSAP) (HELCOM 2021). Scenario B illustrates the results when BSAP is put into effect exclusively within the Archipelago Sea catchment area, aligning with the national abatement objectives outlined in Laamanen et al. (2021). In scenario C, BSAP is implemented in all other regions draining their waters to the Baltic Sea, but not in the catchment area of the Archipelago Sea. In scenario D, BSAP is implemented in all riparian countries of the Baltic Sea.

**Table 1:** Scenarios for nutrient loads originating from the non-point and point sources from its own catchment area, net balance of nutrients from exchange of water with neighboring sea basins, atmospheric deposition, and nutrients released from the sediments of the Archipelago Sea

# Abbreviation	Loads from catchment	Neighbouring sea basins	Atmospheric deposition	Sediment
A BAU	Current	Current	Current	Current
B BAU (BSAP)	Current	BSAP	SSP1	Current
C BSAP (BAU)	BSAP	Current	SSP1	Current
D BSAP	BSAP	BSAP	SSP1	Current
E SSP1	SSP1	SSP1	SSP1	Current
F SSP1+	SSP1+	SSP1+	SSP1+	Current
G GEOENG	Current	Current	Current	Full control
H NOEXTLOAD	Full control	Current	Current	Current
I NO LOCAL LOAD	Full control	BSAP	SSP1	Full control

In BAU (business-as-usual scenario) nutrient loads remain at current level. In BSAP scenario the nutrient reduction targets of Baltic Sea Action Plan (HELCOM 2021) will be achieved. SSP1 and SSP1+ denote future nutrient loads by 2050 and 2100, respectively, which are in accordance with ‘Sustainable Development’ pathway developed by O’Neill et al. (2014) and extended to the nutrient loads to the Baltic Sea by Pihlainen et al. (2020). Full control means complete control of anthropogenic nutrient load (either from sediments, diffuse or point sources)

Scenarios E and F are exploratory scenarios that show the outcomes from the sustainable societal development (SSP1), which is one of the five alternative Shared Socioeconomic Pathways developed by the climate scientists to describe the challenges that climate change creates for climate mitigation and adaptation during the twenty first century (O’Neill et al. 2014). Scenarios E and F represent the nutrient loading in mid-century (year 2050) and by end of the century (2100), respectively as reported in Pihlainen et al. (2020). Scenarios G–I are also explorative scenarios, but they assume technological breakthroughs and higher commitment in the control of diffuse nutrient loading, point sources, and internal loading of nutrients from sediments. Scenarios G and H are variations of the business-as-usual scenario, assuming complete control of anthropogenic nutrient loads from the sediments and catchment area, respectively. Scenario I is an extreme case, assuming complete control of anthropogenic nutrient loads from both sediments and the catchment area of the Archipelago Sea, and implementation of the BSAP elsewhere. Scenarios G–I are utopian given our current technologies but may become plausible in the future as the result of innovations and adoption of yet unforeseen technologies in controlling nutrient loading. The outcomes of these scenarios serve as benchmarks as they represent the highest attainable level of water quality that can be reached with intensive water protection efforts in the region.

### Catchment scale water quality model VEMALA

Scenario narratives and assumptions are used as inputs in a catchment model, which describes the soil and aquatic processes and provides the spatially and temporally explicit projections of nutrient loading from agricultural land, forests, built land, and point sources including wastewater treatment plants and industrial outlets. We used the catchment-scale modeling system VEMALA (Water Quality Watershed Model) to simulate runoff processes, nutrient processes, leaching, and transport on land and in rivers and lakes in Finland (Huttunen et al. 2016, 2021). The VEMALA model provides an estimate for the external loading, outflow loading, and retention of nutrients in all lakes in the catchment area, as well as nutrient loading source apportionment into its main sources: agriculture, forests, scattered settlements, and point sources. VEMALA consists of hydrological sub-model, terrestrial water quality models (Huttunen et al. 2016), field-scale nutrient loading model ICECREAM (Knisel 1993; Tattari et al. 2001) for the simulation of agricultural loading, and the biogeochemical river and lake sub-models (Korppoo et al. 2017). The VEMALA model's validation has been conducted for annual nitrogen (N) and phosphorus (P) loading across Finland's 27 largest river catchments (Huttunen et al. 2016). Additionally, validation has been performed for nine specific river catchments and daily nitrogen concentrations (Huttunen et al. 2021).

### Coastal water quality model FICOS

The outputs from the VEMALA catchment model are used as inputs in a spatially and temporally detailed coastal model that describes the transport and biogeochemical processes in the Archipelago Sea. We used the coastal water quality model FICOS (Lignell et al. 2019; Miettunen et al. 2020) to simulate the nutrient and chlorophyll-*a* concentrations in the Archipelago Sea. FICOS’s nutrient cycling is based on Tyrrell’s (1999) and Kiirikki et al.’s (2001) models with parameterization modifications for the Archipelago Sea (Lignell et al. 2013). The model validation results are available in Lignell et al. (2019). The model produces daily results of nutrient and chlorophyll-*a* concentrations in the modeled area. Chlorophyll-*a* is a widely applied measure of algae biomass and abundance, and it reflects the trophic condition of a waterbody. The water column is divided into a productive layer (ca. top 10 m) and a deep layer. Nutrients are transported vertically between the layers and horizontally between neighboring waterbodies. Sediment nutrient exchange is based on availability of mobile phosphorus. The underlying 3D hydrodynamic model of the Archipelago Sea is an expanded version of the model used in Tuomi et al. (2018) and has a horizontal resolution of 0.25 nautical miles with 40 vertical layers and is nested within a 2 nautical mile, 80 vertical layer Baltic Sea model. The spatial resolution used for biogeochemical modeling in this study matches the national water body management borders in the Archipelago Sea.

### Simulations

We performed the simulations over a 7-year timeframe set in the future, occurring at a point of time between 2050 and 2100. This time frame is selected to account for the assumed socioeconomic developments, technological advancements, and policy changes outlined in the scenario descriptions. It is also a period by which nutrient loading has reached a stable, new equilibrium. The meteorological data from the period 2007–2013 was used to create a representative variation of conditions for both catchment model and the coastal water quality model. With FICOS model, the simulation begins at the beginning of 2006 with initial values for currents and surface height deviation set to zero. Temperature and salinity fields were derived from observational data, and the initial modeled algae biomasses were set to zero to correspond with normal mid-winter conditions. Since the sediment component of FICOS is non-dynamic, the model is relatively insensitive to initial conditions, and one year is sufficient for model spin-up. Climate change scenario associated with nutrient loading is RCP4.5 (Thomson et al. 2011). Global climate circulation model MOHC-HadGEM2-ES and the regional circulation model SMHI-RCA4 were used for the nutrient loading input simulations. Hydrodynamic transport, temperature, and salinity are the same in all scenarios. For results, we consider the spring period to be April and May and the summer period from June to September 7th. The Archipelago Sea model was divided into 95 coastal water bodies (Fig. S7 in Supplementary Material) used to implement national water management and river basin management plans in accordance with the EU Water Framework Directive (Aroviita et al. 2019).

## Results

### Effectiveness of local vs. international nutrient abatement efforts

Based on the scenario simulations the overall average value of April–September chlorophyll-*a* in the Archipelago Sea area ranges from 1.62 to 4.48 μg/l (Table 2). The business-as-usual scenario results in significantly higher chlorophyll-*a* level than the other scenarios, which include policy efforts or socioeconomic developments that reduce nutrient loads. The lowest chlorophyll-*a* levels are reached with the scenario representing the situation with no anthropogenic loading (NO LOCAL LOAD), which sets the highest attainable water quality level. If the BSAP mitigation measures are either implemented only in the Archipelago Sea catchment area [BAU(BSAP)] or only in the other regions draining their waters to the Baltic Sea [BSAP(BAU)] the overall chlorophyll-*a* is 3.55 μg/l or 4.00 μg/l, respectively. However, implementing the BSAP measures in all countries results in significantly lower chlorophyll-*a* average (3.19 μg/l) which is close to the national chlorophyll-*a* GES targets for the Archipelago Sea (ranges from 2.3 to 3 μg/l depending on the area, Laamanen et al. 2021). The simulated chlorophyll-*a* averages for the scenarios representing the yet unseen technological breakthroughs in controlling the diffuse and internal loading (GEOENG and NOEXTLOAD) also lead chlorophyll-*a* levels close to the GES targets (3.37 μg/l and 3.16 μg/l, respectively).

**Table 2:**
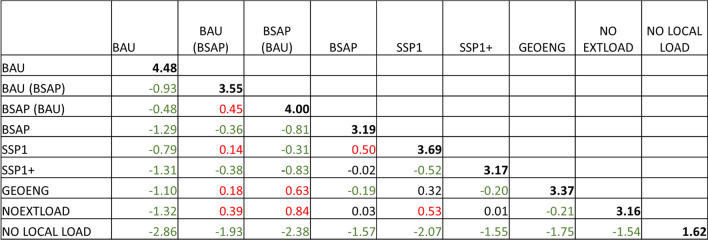
Whole area mean values and mean differences (row–col) in chlorophyll-*a* concentrations between different scenarios (*n* = 97 173)

Diagonal cells (bolded font) are the scenario mean values calculated from the simulated April–September chlorophyll-*a* (µg/l) in surface water layer (0–10 m). Cells below the main diagonal denote the difference in mean values (row–column). Green font indicates a statistically significant negative difference (reduction in chlorophyll-*a*) of paired mean comparisons and green indicates a significant positive difference (increase in chlorophyll-*a*) (*p* < 0.05 *–***). Mean differences and their significances were calculated with Tukey’s HSD test

**Fig. 3:**
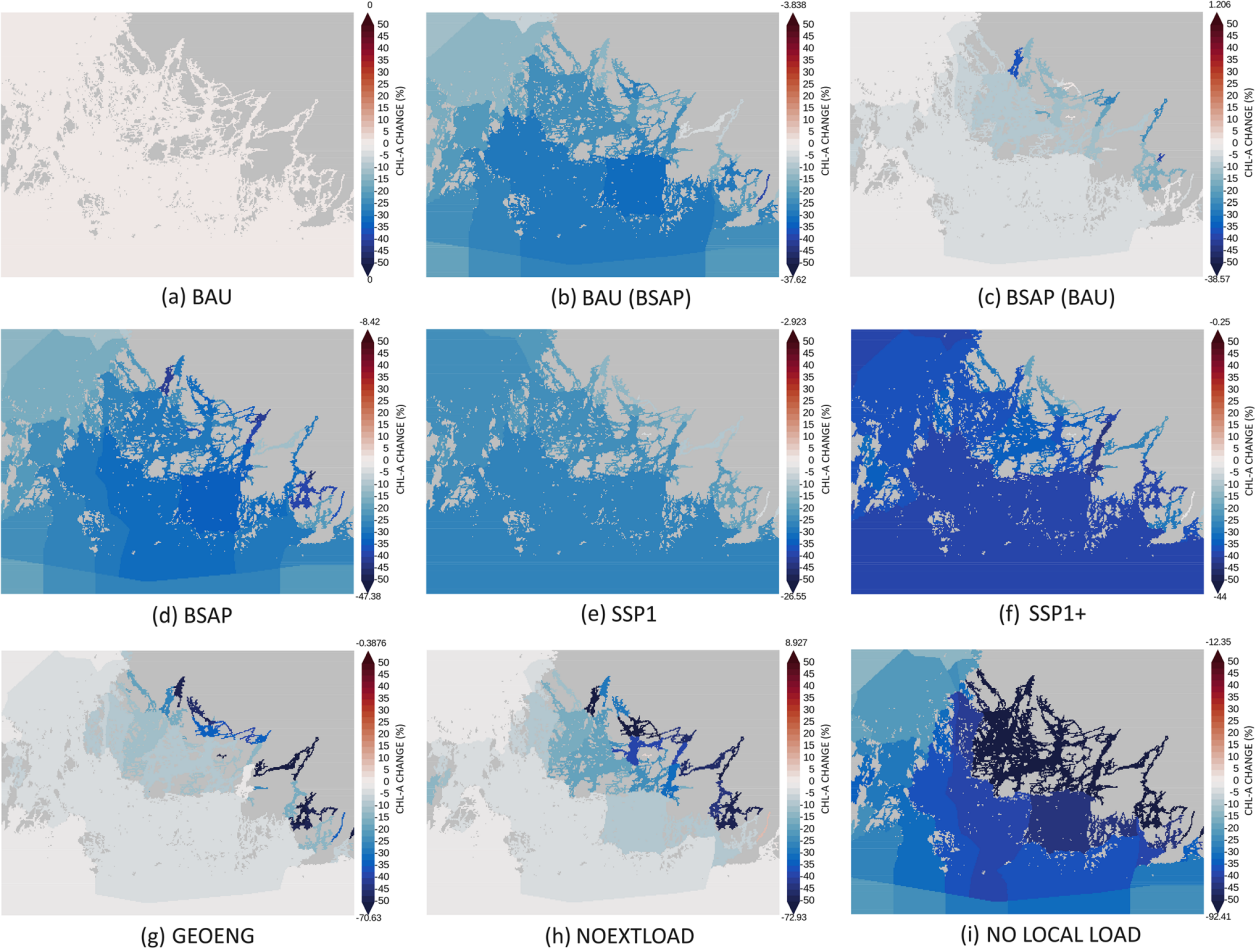
Difference in chlorophyll-*a* concentrations (summer bloom, 1.6–7.9) to business-as-usual scenario in percentages. The highest and lowest values for each of the nine scenarios are shown on both ends of the scale

Reduced loading of nutrients from the catchment area has significant impact on the trophic condition of coastal sea areas around river mouths, estuaries, and innermost sheltered bays (Fig. 3). P concentrations decrease (Fig. S6) but N concentrations may in some areas even increase (Fig. S4). In the strongly P-limited river mouths, reduced P loads lead to a smaller primary production, and consequently, to a less efficient coastal filter for N. However, the simulations also indicate that if the external reductions of N and P from other regions are high enough, the overall N concentrations decrease also in the inner bays.

The impacts of local nutrient abatement efforts gradually decrease as the distance from the coastline or river mouth to the marine area increases and as the water exchange with the open sea increases (Fig. 3). Load reductions from other regions—reflected in this simulation study as changes in the nutrient concentrations of water exchange with neighboring sea areas—have a greater algae-reducing effect than measures in the catchment area. Thus, distance from river mouths and water exchange with open seas determine the relative effectiveness of local nutrient abatement measures. To reach well-balanced improvements in eutrophication status in all parts of the Archipelago Sea, nutrient reductions occurring in the catchment area and other regions are both needed.

### Temporal pattern and magnitude of coastal biomass production

**Fig. 4:**
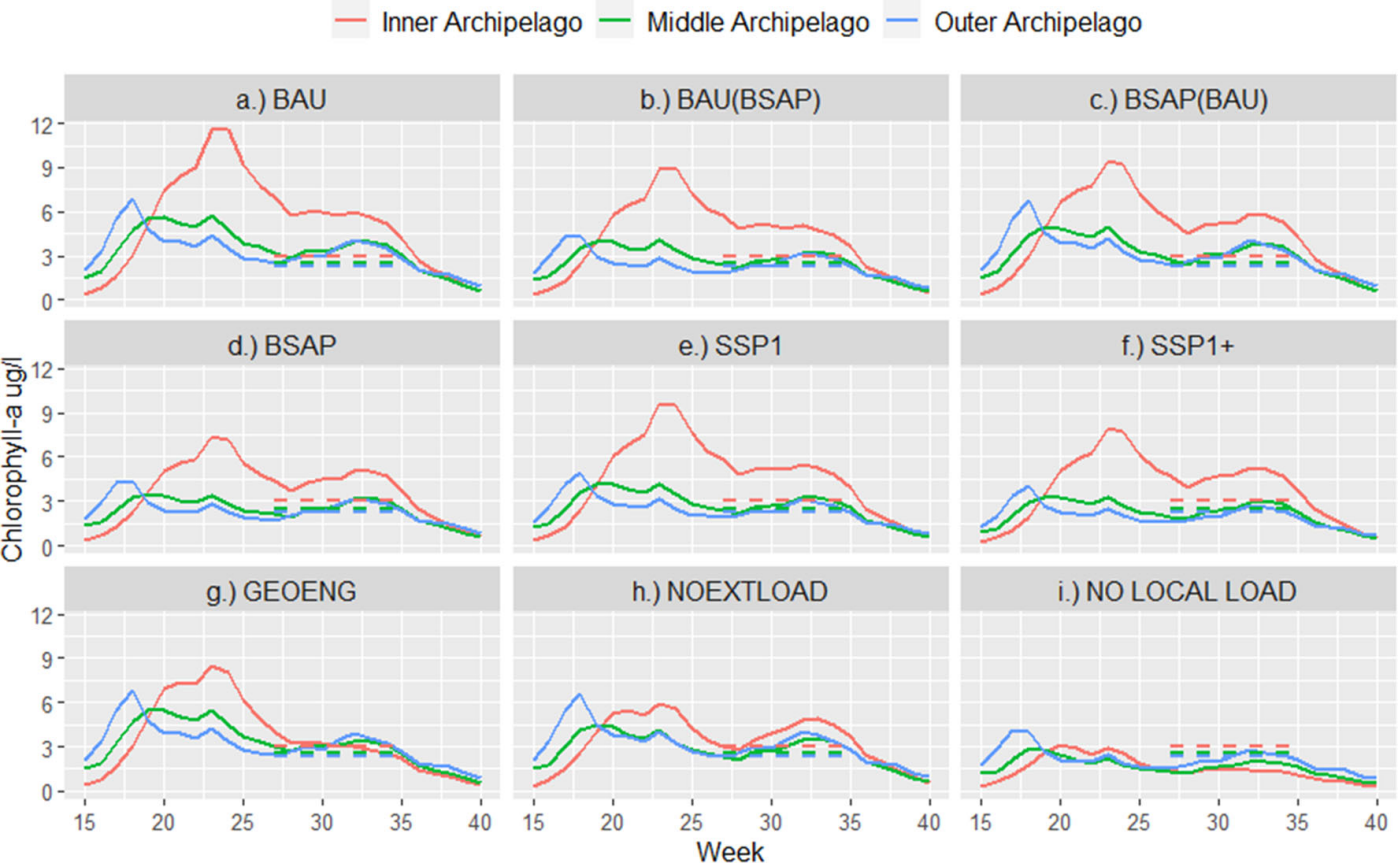
Weekly averages of chlorophyll-*a* concentrations for different scenarios in the inner, middle, and outer archipelago. Dashed horizontal lines denote the national chlorophyll-*a* target values in different parts of the Archipelago Sea

The seasonal variation of chlorophyll-*a* concentrations follows the natural phytoplankton succession with higher biomass peak during the spring (mostly diatoms) and lower peak in late summer (mostly cyanobacteria) (Fig. 4). However, there are differences in the magnitude and timing of the spring peak in different areas and in different management scenarios. In the inner archipelago the spring peak gets very high (weekly max 7.2–11.6 µg/l, depending on the scenario) and starts later (weeks 18–24) than in the outer archipelago where the spring peak occurs less pronounced (weekly max 4.0–6.8 µg/l) and starts earlier (during weeks 17–20). In relation to the GES target values, the simulated June to September chlorophyll-*a* levels exceed the targets in all areas in the BAU scenario (Fig. 4a). The inner archipelago target value (3.0 µg/l) is only reached in the most extreme scenario where the riverine, point source and sediment loading are set to zero (NO LOCAL LOAD Fig. 4i). In this situation, the seasonal pattern is more stable, as the spring and summer peaks are similar in magnitude in all areas. For the outer Archipelago, the most effective scenarios are also those that aim to reduce nutrient loading from neighboring countries along BSAP measures (Fig. 4b–d). These results indicate that anthropogenic disturbance in terms of increased nutrient inputs changes the volume and timing of phytoplankton biomass occurrence.

### Scenario outcomes in comparison to policy goals

**Fig. 5:**
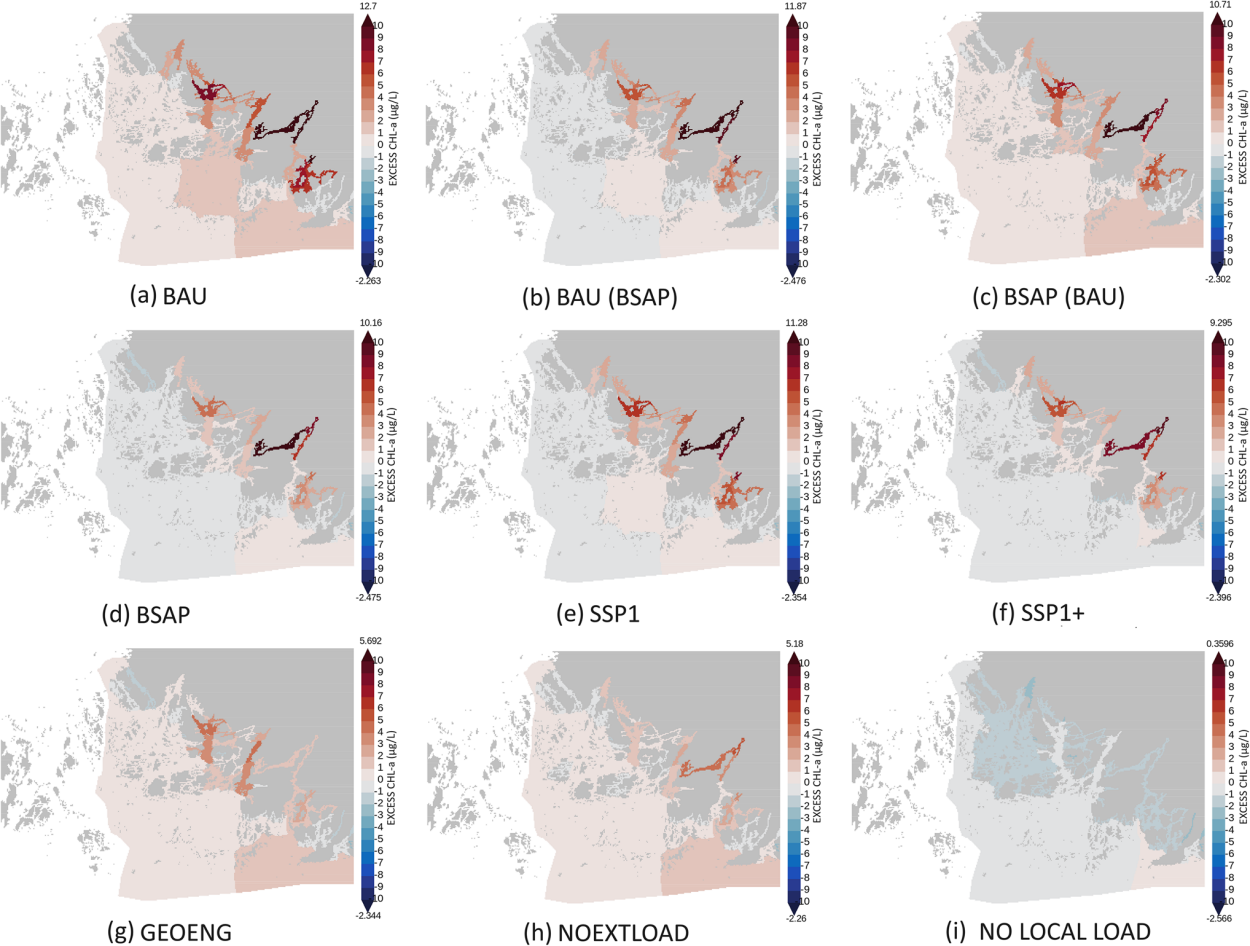
Excess chlorophyll-*a* (µg/l) to the threshold level indicating good ecological status in all scenarios. The concentrations are measured as the average summertime concentration

Most waterbodies are far from reaching good status with the business-as-usual (BAU) scenario (Fig. 5a). Sticking to current nutrient reduction targets in line with the HELCOM Baltic Sea Action Plan (Fig. 5d), the GES threshold can be reached in most areas. However, the threshold is not reached in several enclosed bays in the inner and middle archipelago. With the extreme nutrient abatement scenario (no anthropogenic loading from the catchment area), all areas clearly meet the requirements for nutrient concentrations (Fig. 5i). Thus, it is possible to reach good eutrophication status also in the inner archipelago, but this would require highly effective water protection efforts and the application of new or currently only pilot-tested ecological engineering technologies.

### Areas requiring special attention

**Fig. 6:**
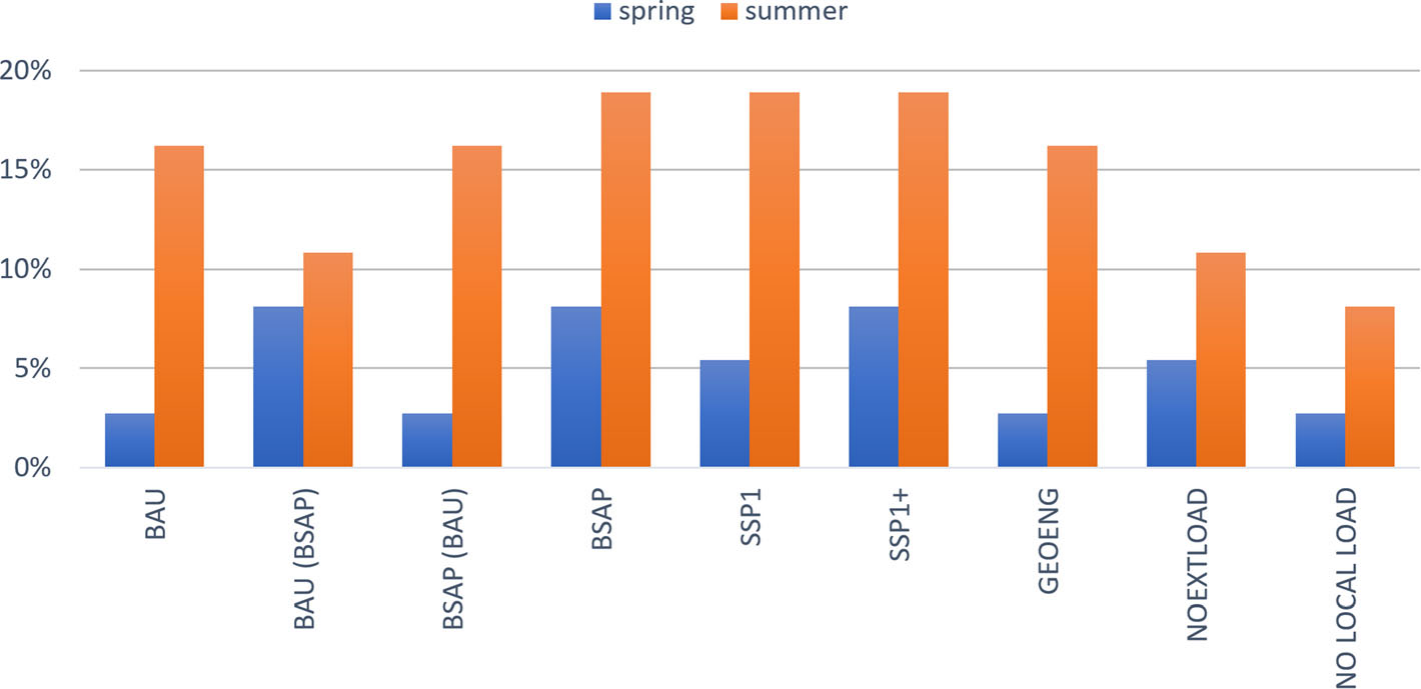
Percentage of waterbodies in the inner archipelago zone where the spring or summer chlorophyll-*a* concentrations exceed the average concentration of all waterbodies in the zone by at least 50%

In addition to averages, information on the temporal and spatial variation of algae biomasses between waterbodies and over the entire year is relevant for many uses of coastal waters including recreation, water sports, and fisheries. Analyzing the simulated responses to water protection efforts may help identify target areas that merit additional water protection efforts. Higher-than-average algae biomasses can result from geomorphological characteristics of waterbody, but carefully targeted measures could improve the state of water in these areas. This is especially important in summertime when phytoplankton is dominated by cyanobacteria harmful for human and animal health. Figure 6 shows the shares of waterbodies in the inner Archipelago where chlorophyll-*a* concentrations exceed the average concentration of all waterbodies by at least 50% separately for summer period (June–August) and spring period (April–May). The share of high concentration waterbodies during the summer is in most scenarios between 10 and 20%, but in spring, the waterbodies behave more uniformly with a share of 8% or less having 50% higher concentration in all scenarios.

The extreme mitigation scenario (with no anthropogenic nutrient loading) shows the smallest variability in chlorophyll-*a* (and smallest number of distinctly deviating waterbodies) both in spring and in summer. This scenario can be considered to reflect natural or undisturbed conditions. In all remaining scenarios, the share of clearly deviating (1.5 × higher chlorophyll-*a* concentration than the mean) waterbodies remain high, and in fact, increases at higher ambition levels of nutrient abatement due to the increased relative share of internal P loading. In other words, the number of problematic waterbodies that require additional effort and consideration remains high even in the most ambitious nutrient abatement scenarios unless internal loading of P from sediments is successfully controlled. Internal loading originates from a pool of P accumulated in the sediments during earlier periods of high external loading. The problem is localized in semi-enclosed coastal waterbodies, including bays where the exchange of water with other sea areas is limited (Fig. S7). Large temporal and spatial variation in water quality indicators challenges the ongoing practice of using common ecosystem GES thresholds for large coastal areas and of using fixed dates for reaching the target.

## Discussion

We combined exploratory and target-seeking scenarios with spatially and temporally explicit models for a coastal sea area and its catchment area to study the effectiveness of local water protection measures for protecting a highly heterogenous archipelago. The model simulations allowed us to better understand the extent and adequacy of current and planned nutrient mitigation efforts and analyze the relevance of currently used indicators. Using the Archipelago Sea as an example, we demonstrated that good eutrophication status is far from attainable in any sea area, nearby coastal region, or inner bay through unilateral local action conducted solely in the drainage basin. We also showed that through coordinated load reductions (joint action) between all the Baltic Sea countries (BSAP or higher efforts), good eutrophication status can be achieved in the Archipelago Sea except for the inner archipelago, river mouths, and inner bays. However, improving all coastal areas, including the inner bays, to a good eutrophication status would require extreme efforts that virtually stop the anthropogenic nutrient load from forests, agricultural land, and municipal and industrial wastewater.

Reaching good eutrophication status in all parts of the Archipelago Sea would require adoption of ecological engineering methods such as pumping oxygen-rich water to anoxic sea bottoms or stopping the sediment P flow by adding materials or chemicals to the sediment surface, to reduce the internal nutrient loading from sea bottoms (Ollikainen et al. 2016; Stigebrandt 2018). Achieving such an outcome would require substantial investments in research and development, and infrastructure both on land and at sea—and willingness of the local society to largely adopt those measures that are found to be effective and are suited for the area. It also eventually requires societal readiness to accept failures and to continue searching for better cost-effective measures. In the long run, a sustainability transition in food production—which has been called for to combat climate change and biodiversity loss (El Bilali et al. 2021)—would also help to reach water protection goals. For example, prospective developments in cellular food (e.g., Klerkx and Rose 2020) would reduce the need for fodder production and would lead to a reduction in agricultural land, thus, facilitating water protection.

When interpreting our results, it is important to emphasize that while physiochemical attributes (such as nutrient concentrations) and biomass (specifically, chlorophyll-*a* levels) provide insights into the eutrophication status, they represent just one facet of the overall ecological state of coastal waters. Several indicators reflecting the health and abundance of plant and animal communities, including the brackish water benthic index (Perus et al. 2007), and the abundance and growth depth of the uniform bladder wrack (*Fucus vesiculosus*) populations, have been developed to assess the ecological state of coastal waters (Aroviita et al. 2019). In many sea areas, however, monitoring is still based on a few key variables: total N, total P, and phytoplankton concentration (chlorophyll-*a* as a proxy) (Andersen et al. 2016; Friedland et al. 2021). Easily observable chlorophyll-*a* is still the main indicator of ecological state in Finnish coastal waters (78% of the waterbodies in the latest 2021 assessment). Measuring environmental quality using indicators that only reflect one aspect of the food web, i.e., primary production, may lead to misjudgments in true ecosystem resilience, wrong management advise, and in the non-optimal allocation of nature protection budgets. For example, it may be economically and ecologically justified in some cases to place larger emphasis on controlling fisheries and hunting—thus, improving the ecosystem’s capability of making use of the increased primary production through better balance between predators and prey—rather than attempting to reach an overly ambitious nutrient abatement goal (Niiranen et al. 2012). Unrealistically ambitious goals may also lead to underperformance if they discourage action and lead to excessively low resourcing of water protection and subsequent losses and reductions in the provision of coastal ecosystem services (Hobbs 2007).

By connecting scenarios and models that describe soil processes within the catchment and biogeochemical processes within the receiving water body, we were able to investigate how existing and prospective policies, as well as potential technological advancements, may impact the characteristics of the specific sea area under examination. Our modeling approach can be applied to similar semi-enclosed coastal areas and inland lakes, offering replicability for further research. However, we note that there are several areas in our modeling framework that could be further improved. First, the models used in this study only partially consider the effects of a changing climate. While they account for climate-induced impacts on soil processes and nutrient runoff from the catchment area, they do not incorporate changes in the physical characteristics of water (temperature, salt concentrations) in the receiving water body. Additionally, the current version of the biogeochemical model employs a static sediment storage component. A dynamic version of the model would yield more realistic insights into the temporal patterns of phosphorus release from the sediments. Furthermore, the integration of ecosystem models within the modeling framework would offer several advantages, including enhanced indicators and a more cohesive assessment of the ecological condition.

The Baltic Sea ecosystem has been in transition since the end of the last glacial period 10 000 years ago. In recent decades, climate change and multiple anthropogenic pressures have emerged as new catalysts of change (Reusch et al. 2018). Our simulations demonstrate how the seasonal timing of phytoplankton biomass occurrence and magnitude changes along with anthropogenic disturbance. A phenological change has been particularly considered a broad-scale indicator of climate change (Edwards and Richardson 2004; Kharouba et al. 2018). However, temporal shifts in phytoplankton occurrence have also been connected to changes in more local processes such as nutrient status and eutrophication (Suikkanen et al. 2013; Desmit et al. 2020). As load reductions decrease nutrient reserves in the water mass outside the growth season, they have a direct effect on the spring bloom period, which utilizes these nutrient reserves. In most of the study area, dissolved nitrogen is limiting nutrient for spring bloom. Load reductions have only an indirect control on the summer productivity, which makes its target setting difficult as we still have limited understanding, e.g., on regenerative nutrient processes. These are dependent on temperature regime and organic carbon availability, which make generalization difficult at this moment. Furthermore, climate change will influence the whole growth period mainly by starting temperature stratification earlier in spring and increasing summer temperature. Both these phenomena have consequences on phytoplankton succession by earlier spring bloom and mostly unknown effect on summer algal communities. Phytoplankton spring bloom intensity has already been suggested as an indicator of eutrophication (Fleming and Kaitala 2006). Our results support the importance and possibilities of assessing the spring bloom timing and magnitude. As phytoplankton plays an important role in the pelagic food-web energy transfer, the temporal shifts in the occurrence and magnitude of chlorophyll-*a* may lead to changes at higher trophic levels and eventually to ecosystem-level changes (Falkowski et al. 1998; Casini et al. 2008). Changes are also expected in the timing of nutrient loading peaks. Simulation studies have shown that an increasing share of nutrient loading will occur during the winter season when soils are prone to nutrient leaching due to erosion (Huttunen et al. 2015, 2021). Understanding such changes, transitions, and interactions is necessary for planning mitigation measures to reduce unnecessary stress and build the resilience of coastal ecosystems.

One way to improve the resilience of coastal ecosystems under changing conditions is to develop spatially and temporally targeted management actions that account for variations in the vulnerability of receiving coastal ecosystems and the expected climate change-driven shifts in the timing of nutrient loading and phytoplankton peaks. Spatially targeted measures are easier to implement, as they can be focused on those catchments that drain into the most sensitive coastal seas. Opportunities for temporally targeted nutrient control that timely cut the concentrations of nutrients before peak phytoplankton periods are more limited. For example, artificial oxygenation would reduce the internal loading of P from the sea sediments to feed in P-limited summer bloom, but its impact is temporary and a number of risks are associated with the procedure (Conley et al. 2009; Ollikainen et al. 2016).

Our simulations support the general understanding and demonstrate that both local water protection measures and measures conducted elsewhere are necessary to achieve improvements in local water quality even in a relative closed Archipelago Sea (Figs. 3 and 5). Obtainable synergies encourage co-operation across regions and between neighboring countries sharing the coastline of coastal seas. Achieving good eutrophication status (including in the archipelago and the country's own territorial waters) will only succeed if neighboring regions and countries commit to reductions or if parallel societal developments lead to reductions in nutrient loading. Ingredients for success include multilateral environmental agreements that are mutually beneficial for all participants (Wagner 2001), the existence of international organizations taking care of the governance of regional seas (Van Tatenhove 2013), a smooth transfer of knowledge and technologies across sectors, regions, and countries (Vierros and Harden-Davies 2020), and stakeholder involvement in marine protection and governance (Morf et al. 2019).

### Supplementary Information

Below is the link to the electronic supplementary material.

Supplementary file1 (PDF 2959 KB)
