# Supplementary material for: Good eutrophication status is a challenging goal for coastal waters
Source: Ambio. 2023 Dec 23;53(4):579–91. doi: 10.1007/s13280-023-01965-7 (PMC10920530; doi:10.1007/s13280-023-01965-7)
Supplement: Supplementary file 1 — Supplementary file1 (PDF 2959 KB) [file 13280_2023_1965_MOESM1_ESM.pdf]

Ambio

Electronic Supplementary Material

This supplementary information has not been peer reviewed.

Title: Good eutrophication status is a challenging goal for coastal waters

Authors: Kari Hyytiäinen, Inese Huttunen, Niina Kotamäki, Harri Kuosa, Janne Ropponen

Nutrient loads in different scenarios

Tables S1-S5 show the nutrient loads and concentrations relative to their current levels in all 9 studied scenarios. Changes in nutrient loading in other areas are reflected as changes in the nutrient concentrations of sea water in the northern, eastern, southern, and western boundaries of the Archipelago Sea basin. In addition to the riverine loading and exchange of water and nutrient with adjacent sea basins, the levels of atmospheric deposition, direct point source loads and internal loading of phosphorus are determined.

Nutrient loads of Business-as-usual scenario (BAU) are average loads from the period 2007-2013, and they are based on measurements and validated models.

The daily catchment loading inputs from VEMALA are distributed to relevant sea locations, e.g. river mouths. The atmospheric loading of nitrogen and phosphorus are distributed evenly spatially and have a monthly distribution. Internal loading estimates are based on an empirical–statistical model and contain both spatial and monthly variation. The annual sum of both atmospheric and internal loading remains constant over time.

Table S1 Riverine nutrient loading

Scenario	DIN (tn/a)	DIP (tn/a)	TN (tn/a)	TP (tn/a)	DIN%	DIP%	TN%	TP%
BAU	4575	126	6958	420	100.0 %	100.0 %	100.0 %	100.0 %
BAU (BSAP)	4575	126	6958	420	100.0 %	100.0 %	100.0 %	100.0 %
BSAP (BAU)	3954	87	7518	297	86.4 %	69.0 %	108.0 %	70.7 %
BSAP	3954	87	7518	297	86.4 %	69.0 %	108.0 %	70.7 %
SSP1	3701	92	5629	304	80.9 %	73.0 %	80.9 %	72.4 %
SSP1+	2688	63	4090	215	58.8 %	50.0 %	58.8 %	51.2 %
GEOENG	4575	126	6958	420	100.0 %	100.0 %	100.0 %	100.0 %
NOEXTLOAD	1180	29	1795	96	25.8 %	23.0 %	25.8 %	22.9 %
NO LOCAL LOAD	1180	29	1795	96	25.8 %	23.0 %	25.8 %	22.9 %

Table S2. Atmospheric deposition

Scenario	DIN (tn/a)	DIP (tn/a)	TN (tn/a)	TP (tn/a)	DIN%	DIP%	TN%	TP%
BAU	4396	81	4396	81	100.0 %	100.0 %	100.0 %	100.0 %
BAU (BSAP)	3095	81	3095	81	70.4 %	100.0 %	70.4 %	100.0 %
BSAP (BAU)	3095	81	3095	81	70.4 %	100.0 %	70.4 %	100.0 %
BSAP	3095	81	3095	81	70.4 %	100.0 %	70.4 %	100.0 %
SSP1	3095	81	3095	81	70.4 %	100.0 %	70.4 %	100.0 %
SSP1+	2467	81	2467	81	56.1 %	100.0 %	56.1 %	100.0 %
GEOENG	4396	81	4396	81	100.0 %	100.0 %	100.0 %	100.0 %
NOEXTLOAD	4396	81	4396	81	100.0 %	100.0 %	100.0 %	100.0 %
NO LOCAL LOAD	3095	81	3095	81	70.4 %	100.0 %	70.4 %	100.0 %

The atmospheric deposition of nitrogen is a significant source of nitrogen in the Baltic Sea region. It consists of the emissions of nitrates (NO_x) mainly originating from traffic and energy production in central Europe, and emissions of ammonia (NH_x) mainly originating from local sources, and in particular from animal husbandry. The NO_x emissions have been projected to decrease in northern Europe for most socio-economic scenarios (Rao et al. 2017), while NH_x emission largely depend on the magnitude of animal husbandry and the technologies used in storing and handling animal manure. Given the south-western winds characteristic of the study area, current fast transition away from using fossil fuels in traffic and energy production, and recent designation of the Baltic Sea as an emission control area for nitrogen oxides (NECA) we believe that atmospheric deposition of nitrogen decreases to the level shown by the SSP1 scenario also with other policy scenarios (Pihlainen et al. 2020).

Table S3. Internal loading

Scenario	DIN (tn/a)	DIP (tn/a)	TN (tn/a)	TP (tn/a)	DIN%	DIP%	TN%	TP%
BAU	3795	3563	3795	3563	100.0 %	100.0 %	100.0 %	100.0 %
BAU (BSAP)	3795	3563	3795	3563	100.0 %	100.0 %	100.0 %	100.0 %
BSAP (BAU)	3795	3563	3795	3563	100.0 %	100.0 %	100.0 %	100.0 %
BSAP	3795	3563	3795	3563	100.0 %	100.0 %	100.0 %	100.0 %
SSP1	3795	3563	3795	3563	100.0 %	100.0 %	100.0 %	100.0 %
SSP1+	3795	3563	3795	3563	100.0 %	100.0 %	100.0 %	100.0 %
GEOENG	0	0	0	0	0.0 %	0.0 %	0.0 %	0.0 %
NOEXTLOAD	3795	3563	3795	3563	100.0 %	100.0 %	100.0 %	100.0 %
NO LOCAL LOAD	0	0	0	0	0.0 %	0.0 %	0.0 %	0.0 %

Internal loading is caused by large phosphorus stocks that accumulated in the sea bottom sediments during the several decades of high nutrient pollution in the 1950-1990. Currently, a proportion of this phosphorus stock is released back to the water column during the periodic anoxic conditions at the sea bottom. In shallow sea areas - characteristic of the Archipelago Sea - the internal loading of phosphorus is a result of temperature stratification of water during the summer. Stratification prevents the exchange of water below and above the thermocline and can lead to anoxic conditions if the oxygen stocks are consumed due to excessively high biological production and biodegradation of the died algae biomass. However, as the result of significantly reduced external nutrient loading, the biological production and decomposition remains at the level not exceeding the oxygen stocks during May-September.

Table S4. Direct point source loading

Scenario	DIN (tn/a)	DIP (tn/a)	TN (tn/a)	TP (tn/a)	DIN%	DIP%	TN%	TP%
BAU	859	25	937	64	100.0 %	100.0 %	100.0 %	100.0 %
BAU (BSAP)	859	25	937	64	100.0 %	100.0 %	100.0 %	100.0 %
BSAP (BAU)	859	25	937	64	100.0 %	100.0 %	100.0 %	100.0 %
BSAP	859	25	937	64	100.0 %	100.0 %	100.0 %	100.0 %
SSP1	859	25	937	64	100.0 %	100.0 %	100.0 %	100.0 %
SSP1+	859	25	937	64	100.0 %	100.0 %	100.0 %	100.0 %
GEOENG	859	25	937	64	100.0 %	100.0 %	100.0 %	100.0 %
NOEXTLOAD	0	0	0	0	0.0 %	0.0 %	0.0 %	0.0 %
NO LOCAL LOAD	0	0	0	0	0.0 %	0.0 %	0.0 %	0.0 %

Data on point loading have been obtained from the Finnish Environmental Administration's environmental permit system and database VAHTI (SYKE 2022), which contains all nutrient sources within the study area, including wastewater treatment plants, industry, and fisheries. Monthly variation and fractions of bioavailable nutrients have been estimated separately for each nutrient source.

Table S5. Boundary loads (in percentages of the BAU scenario)

	Total Nitrogen				Total Phosphorus			
Scenario	North	East	South	West	North	East	South	West
BAU	100.0 %	100.0 %	100.0 %	100.0 %	100.0 %	100.0 %	100.0 %	100.0 %
BAU (BSAP)	104.5 %	44.2 %	58.8 %	69.4 %	104.5 %	61.7 %	39.4 %	46.7 %
BSAP (BAU)	100.0 %	100.0 %	100.0 %	100.0 %	100.0 %	100.0 %	100.0 %	100.0 %
BSAP	100.0 %	44.2 %	58.8 %	69.4 %	100.0 %	61.7 %	39.4 %	46.7 %
SSP1	81.0 %	73.0 %	73.0 %	73.0 %	69.0 %	83.0 %	83.0 %	83.0 %
SSP1+	60.0 %	61.0 %	61.0 %	61.0 %	53.0 %	77.0 %	77.0 %	77.0 %
GEOENG	100.0 %	100.0 %	100.0 %	100.0 %	100.0 %	100.0 %	100.0 %	100.0 %
NOEXTLOAD	100.0 %	100.0 %	100.0 %	100.0 %	100.0 %	100.0 %	100.0 %	100.0 %
NO LOCAL LOAD	100.0 %	44.2 %	58.8 %	69.4 %	100.0 %	61.7 %	39.4 %	46.7 %

The marine streams and concentrations of nutrients on both sides of the boundary between the study area and neighboring sea basins determine the net flow of nutrients. Within BSAP scenario, the reductions in nutrient concentrations occur in western, southern and eastern boundaries of the study area, while within the northern boundary the concentrations remain the same or slightly increase. The sea basins north of Archipelago Sea (Bothnian Bay and Bothnian Sea) are mostly in good or moderate state and no additional nutrient abatement activities have been planned for the catchment areas draining to these northern basins of the Baltic Sea.

External or boundary loadings were estimated using weekly measured data from the Utö and Brändö intensive monitoring stations for current conditions (HELCOM 2018) and nutrient loading under the current target documented in the Baltic Sea Action Plan (HELCOM 2013).

Table S6. Compilation of the sources of nutrient loading to the Archipelago Sea from Finland.

	Phosphorus		Nitrogen	
	Tons	%	Tons	%
Municipalities	9	2	430	7
Industry	<1	<1	14	<1
Aquaculture	13	4	134	2
Peat industry	<1	<1	2	<1
Agriculture	320	87	4400	67
Dispersed settlement	22	6	160	2
Forestry	3	1	100	1
Runoff water	<1	<1	19	<1
Atmospheric input	n.a.	n.a.	560	9
Shipping	n.a.	n.a.	660	10

The figures are given as tons y^{-1} and percentages on total phosphorus and total nitrogen loads. Diffuse loading is the average of 2010 to 2019 and point sources the value of 2019. Loading from ships is calculated for the Archipelago Sea from the average in the total loading to the Finnish marine waters from 2012 to 2017.

Supplementary results

The main outcomes of simulations are visualized as maps in Figures S1-S7. Figure S1 shows the average chlorophyll concentrations for all studied 9 scenarios. Differences in chlorophyll concentrations in comparison to the business-as-usual scenario are shown for spring bloom in Figure S2 (see Fig 3 in the main text for results during summer). Figures S3 and S5 show average nitrogen and phosphorus concentrations, and Figures S4 and S6 show the distances in concentrations in comparison to business-as-usual scenario. Figure S7 shows a map of hot spot waterbodies that would require additional and targeted measures even if the current nutrient reduction targets (articulated in the Baltic Sea Action Plan) were fully reached.

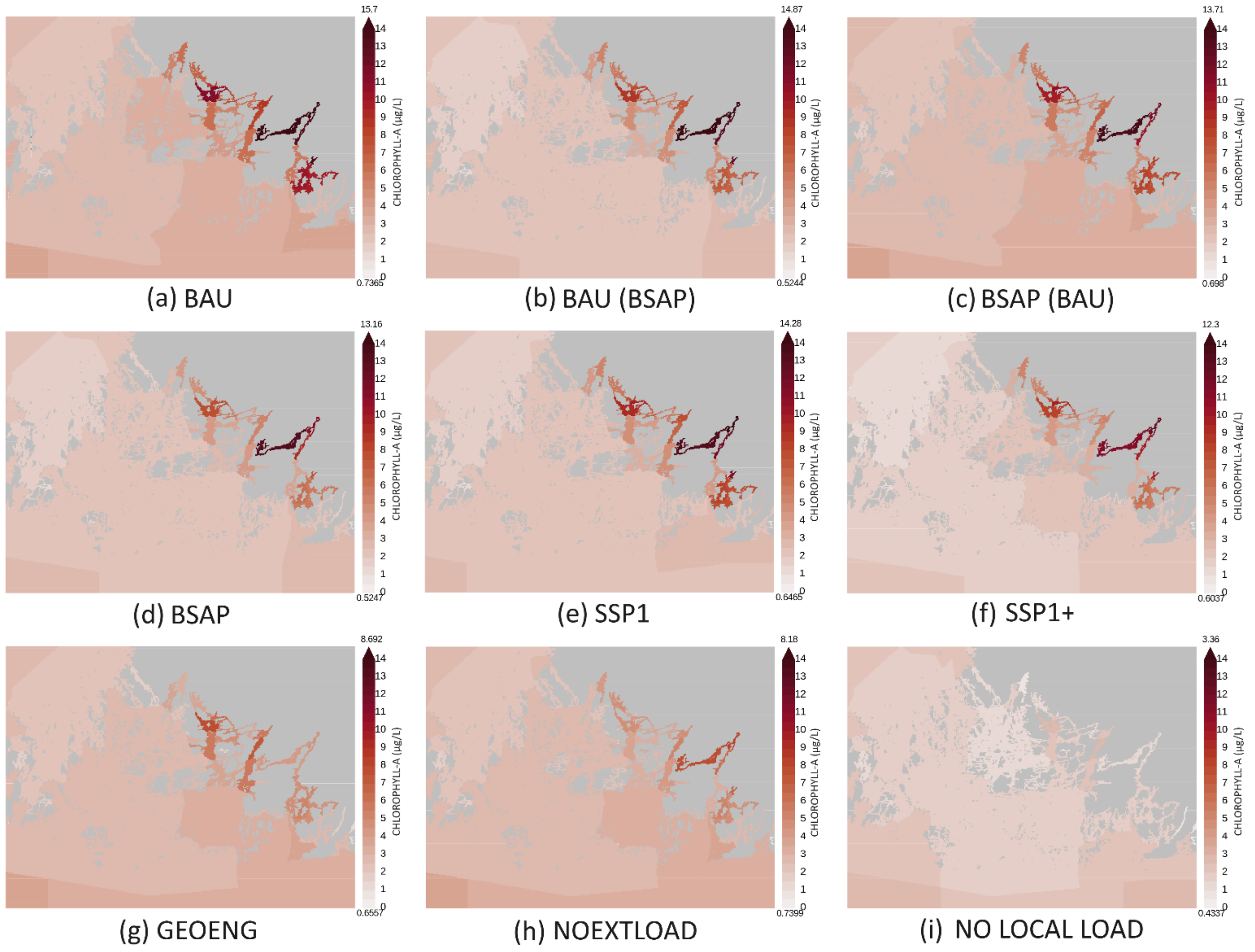

Fig. S1. Average chlorophyll-a concentrations (µg/l) in surface water (at depths 0-10 m) during the summer period: 1. June - 7. September. The highest and lowest values for each of the 9 scenarios are shown on both ends of the scale.

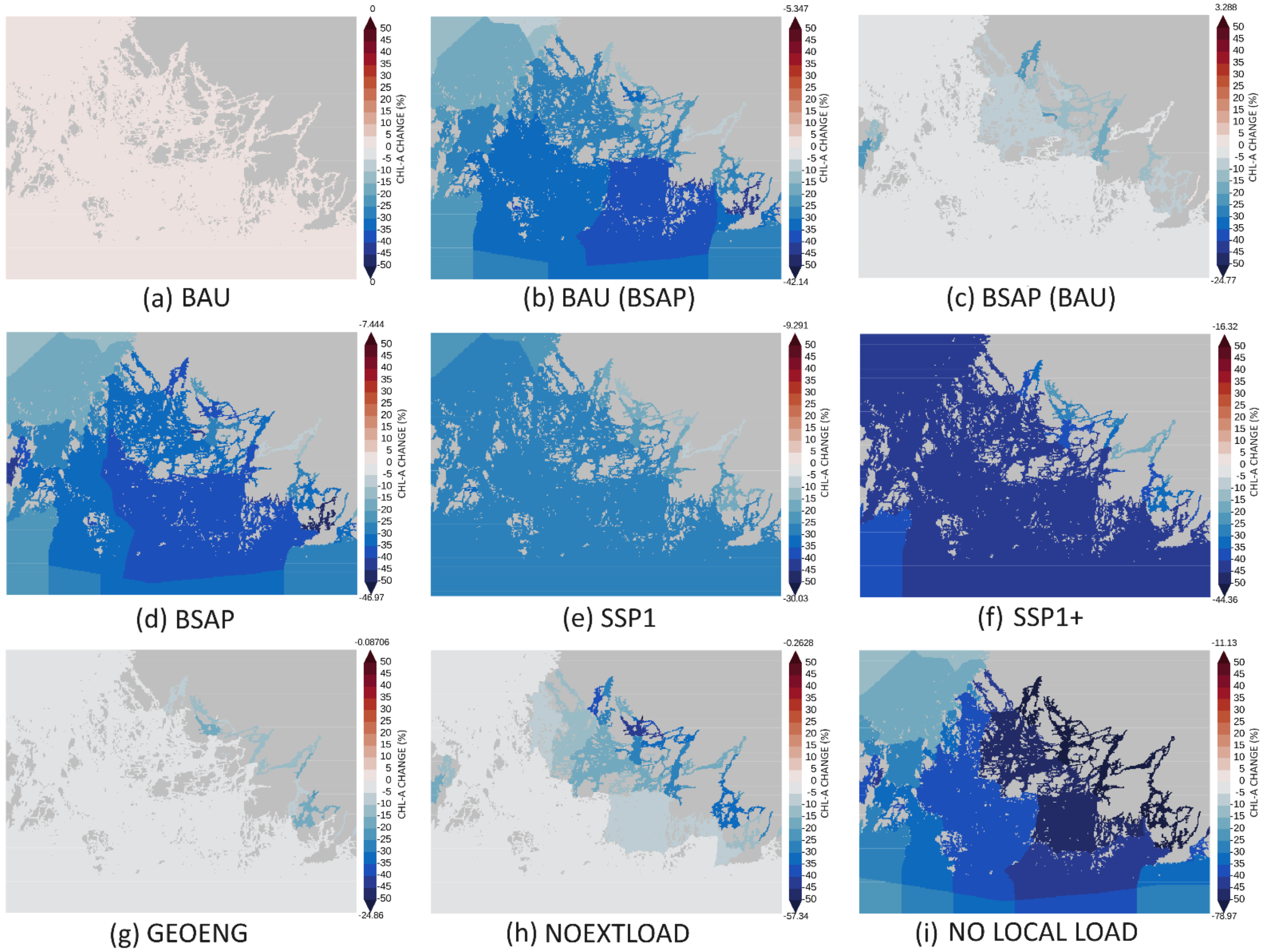

Fig. S2. Difference in chlorophyll-a concentrations (spring bloom, 1.4-31.5) to business as usual-scenario in percentages. The highest and lowest values for each of the 9 scenarios are shown on both ends of the scale.

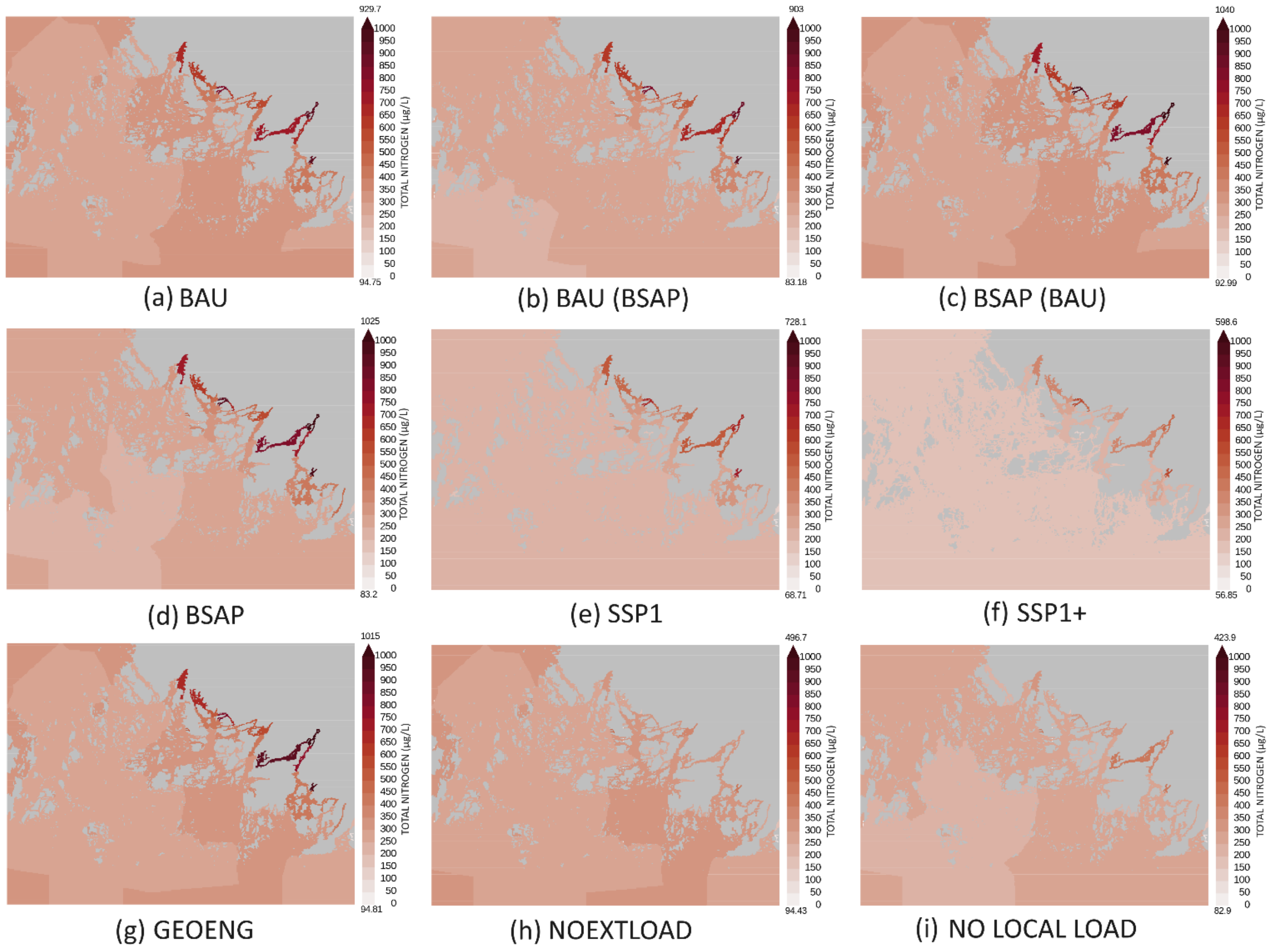

Fig. S3. Average nitrogen concentrations in surface water (µg/l) during entire year

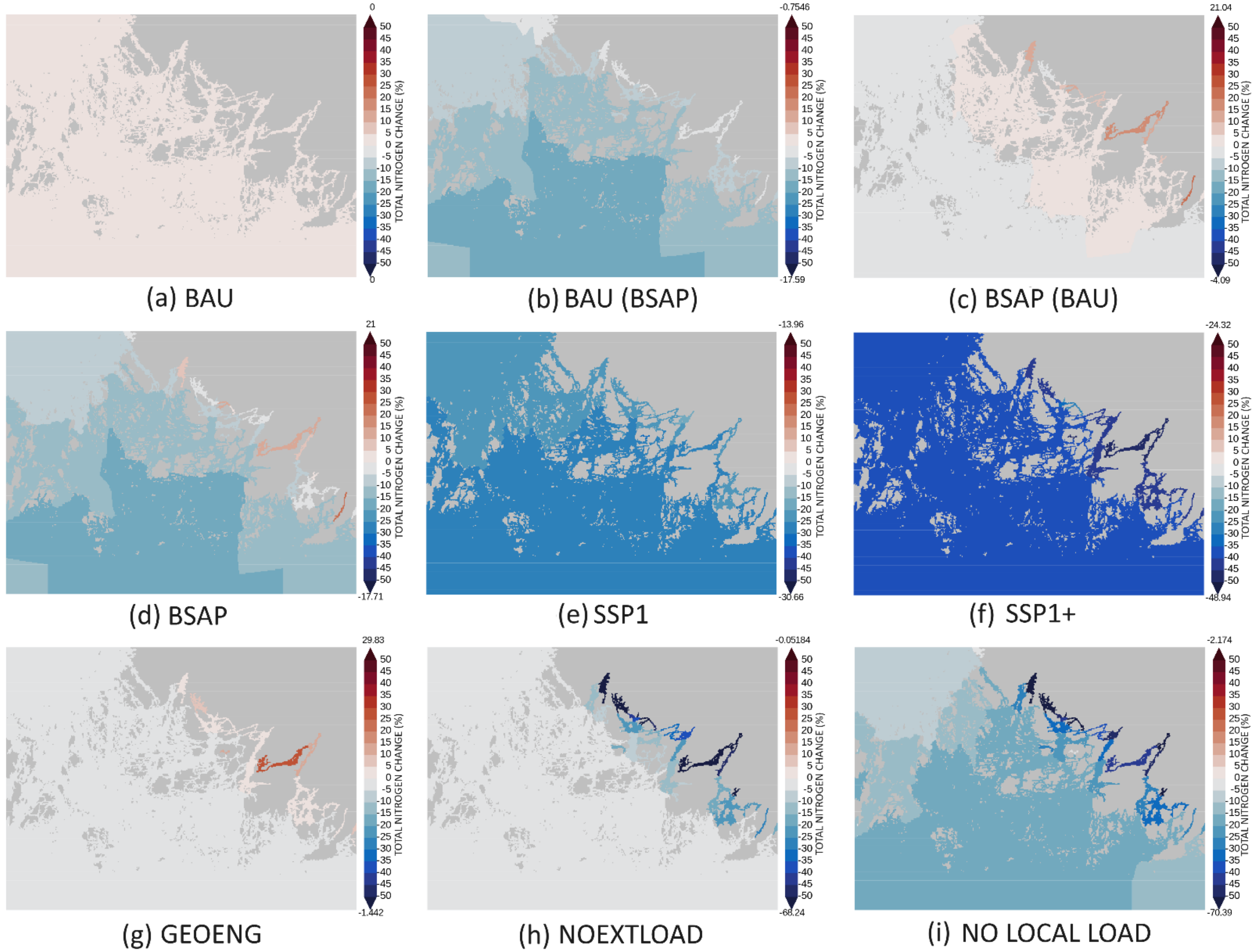

Fig. S4. Difference in surface nitrogen concentrations compared to the business-as-usual scenario in percentages

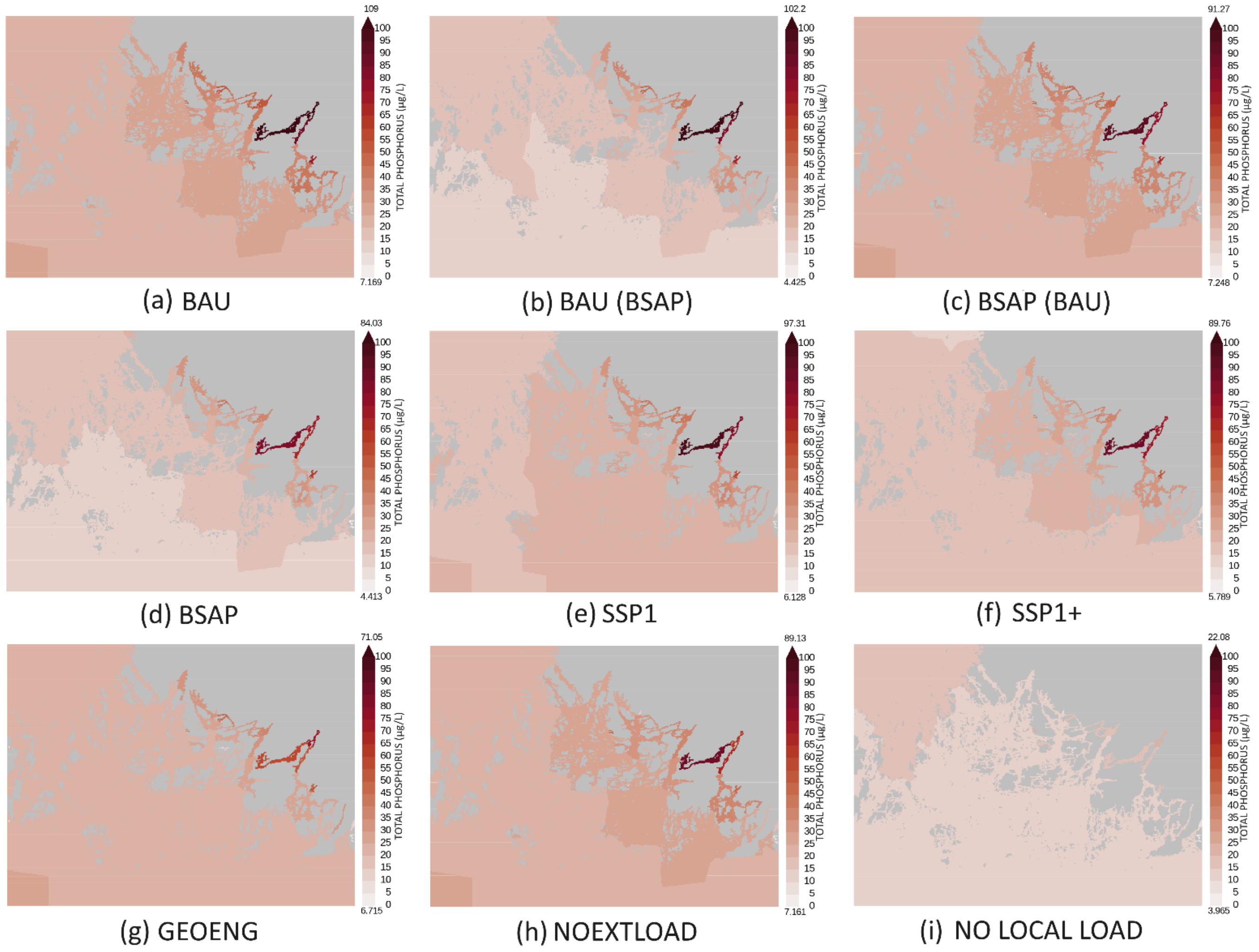

Fig. S5. Average phosphorus concentrations in surface water (µg/l) during entire year

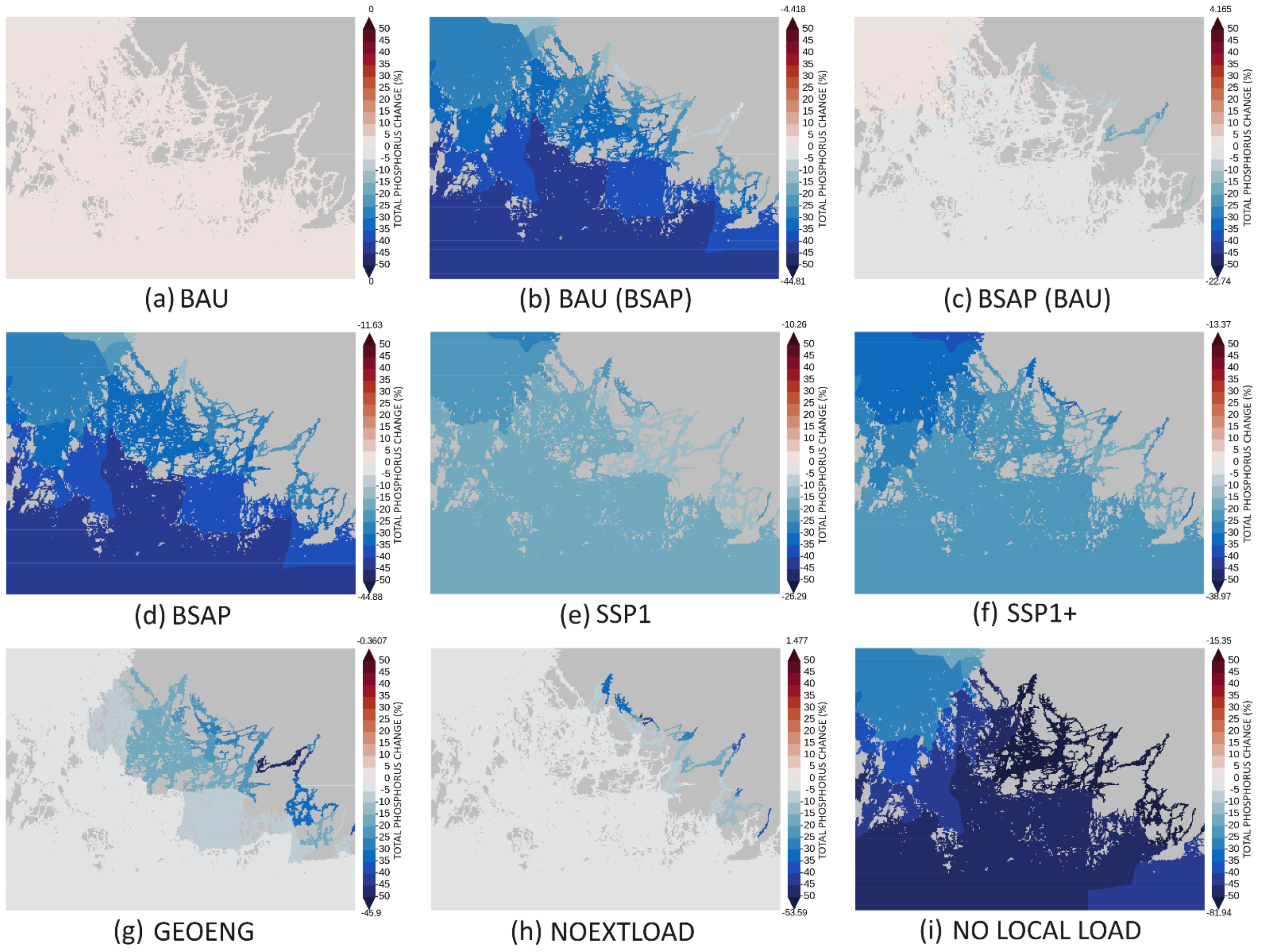

Fig. S6. Difference in surface phosphorus concentrations compared to the business-as-usual scenario in percentages

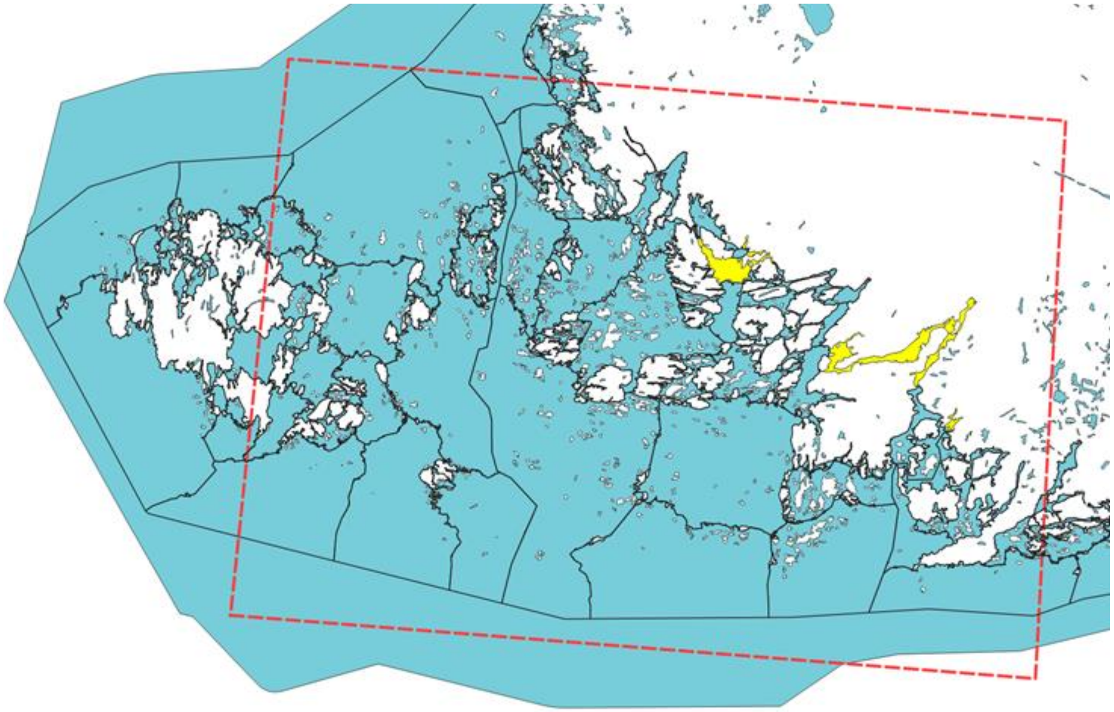

Fig S7. The 7 inner archipelago hot spot waterbodies (in yellow) requiring additional and targeted measures, even if the Baltic Sea Action has been implemented. The waterbodies have a summer period average chlorophyll-a concentration that is over 1.5 times that of the average chlorophyll-a concentration of all the inner archipelago water formations.

SI REFERENCES

HELCOM. 2013. Approaches and methods for eutrophication target setting in the Baltic Sea region.*in* Baltic Sea Environment Proceedings. Helsinki Commission Press Helsinki, Finland.

HELCOM. 2018. State of the Baltic Sea–Second HELCOM holistic assessment 2011–2016. Pages 1-155 *in* Baltic Sea Environment Proceedings.

Pihlainen, S., M. Zandersen, K. Hyytiäinen, H. E. Andersen, A. Bartosova, B. Gustafsson, M. Jabloun, M. McCrackin, H. M. Meier, and J. E. Olesen. 2020. Impacts of changing society and climate on nutrient loading to the Baltic Sea. Science of the Total Environment **731**:138935.

Rao, S., Z. Klimont, S. J. Smith, R. Van Dingenen, F. Dentener, L. Bouwman, K. Riahi, M. Amann, B. L. Bodirsky, and D. P. van Vuuren. 2017. Future air pollution in the Shared Socio-economic Pathways. Global Environmental Change **42**:346-358.

SYKE. 2022. Open data portal. Finnish Environment Institute.
